# High Expression of *ACE2* and *TMPRSS2* at the Resection Margin Makes Lung Cancer Survivors Susceptible to SARS-CoV-2 With Unfavorable Prognosis

**DOI:** 10.3389/fonc.2021.644575

**Published:** 2021-05-21

**Authors:** Qianqian Wang, Liangyu Li, Tianyu Qu, Jie Li, Lingxiang Wu, Kening Li, Ziyu Wang, Mengyan Zhu, Bin Huang, Wei Wu, Min Wu, Rong Ding, Zhihong Zhang, Qianghu Wang, Xinyi Xia, Pengping Li, Zhi Zhang, Renhua Guo

**Affiliations:** ^1^ Department of Oncology, The First Affiliated Hospital of Nanjing Medical University, Nanjing, China; ^2^ Center for Global Health, School of Public Health, Nanjing Medical University, Nanjing, China; ^3^ Department of Bioinformatics, Nanjing Medical University, Nanjing, China; ^4^ Department of Pathology, The First Affiliated Hospital of Nanjing Medical University, Nanjing, China; ^5^ Institute of Laboratory Medicine, Jinling Hospital, Nanjing University School of Medicine, Southern Medical University, Nanjing, China; ^6^ Department of Laboratory Medicine & Blood Transfusion, Wuhan Huoshenshan Hospital, Wuhan, China; ^7^ Joint Expert Group, Wuhan Huoshenshan Hospital, Wuhan, China; ^8^ Department of Thoracic Surgery, Jiangsu Cancer Hospital and Jiangsu Institute of Cancer Research and Nanjing Medical University Affiliated Cancer Hospital, Nanjing, China

**Keywords:** COVID-19, lung cancer, *ACE2*, single cell, tmprss2

## Abstract

**Background:**

Coronavirus disease 2019 (COVID-19) has rapidly spread worldwide. Systematic analysis of lung cancer survivors at molecular and clinical levels is warranted to understand the disease course and clinical characteristics.

**Methods:**

A single-center, retrospective cohort study was conducted in 65 patients with COVID-19 from Wuhan Huoshenshan Hospital, of which 13 patients were diagnosed with lung cancer. The study was conducted from February 4 to April 11, 2020.

**Results:**

During the course of treatment, lung cancer survivors infected with severe acute respiratory syndrome coronavirus 2 (SARS-COV-2) had shorter median time from symptom onset to hospitalization (*P* = 0.016) and longer clinical symptom remission time (*P* = 0.020) than non-cancer individuals. No differences were observed among indicators such as time from symptom onset to hospitalization and symptom remission time between medium-term and short-term survivors. The expression of *ACE2* (*P* = 0.013) and *TMPRSS2* (*P <*0.001) was elevated in lung cancer survivors as compared with that in non-cancer individuals.

**Conclusions:**

*ACE2* and *TMPRSS2* levels were higher at resection margins of lung cancer survivors than those in normal tissues of non-cancerous individuals and may serve as factors responsible for the high susceptibility to COVID-19 among lung cancer survivors. Lung cancer patients diagnosed with COVID-19, including medium-term survivors, have worse outcomes than the general population.

## Introduction

The outbreak of coronavirus disease 2019 (COVID-19) induced by severe acute respiratory syndrome coronavirus 2 (SARS-COV-2) has rapidly spread around the world (1), affecting more than 214 countries and regions. By July 28, 2020, over 17,000,000 infected cases and 670,000 deaths have been reported worldwide, and the virus continuous to rapidly spread in many countries (COVID-19 Map Johns Hopkins University and Medicine). The World Health Organization (WHO) declared COVID-19 a public health emergency of international concern and announced the current outbreak as a global pandemic.

Coronaviruses are a large family of viruses known to cause diseases ranging from common cold to more severe illnesses such as Middle East respiratory syndrome (MERS) and SARS (2, 3). In comparison with SARS and MERS, COVID-19 exhibits milder clinical impairment but shows a dramatically higher human-to-human transmission rate (4–6). SARS-CoV-2 has been reported to enter cells *via* binding to angiotensin-converting enzyme 2 (*ACE2*) and its co-factor transmembrane protease serine 2 (*TMPRSS2*), which are expressed in the lung and bronchial branches (6). Therefore, the high expression of *ACE2* and *TMPRSS2* in the lung may serve as the molecular mechanism underlying the susceptibility to SARS-CoV-2. The lung is the most frequently targeted organ in lung cancer and COVID-19 pneumonia. Recent studies have demonstrated the aberrant expression of *ACE2* in many tumors and the higher level of *ACE2* in lung adenocarcinoma (LUAD) tissues (7, 8). Lung cancer is the most common cancer and a leading cause of cancer-related death worldwide (9). Surgical resection remains the primary and preferred approach for the treatment of stages I and II non-small cell lung cancer (NSCLC) (10). The expression of *ACE2* and *TMPRSS2* in resection margin tissues, not tumors, of lung cancer survivors is more representative of the susceptibility to SARS-CoV-2.

In the present study, we compared the expression levels of *ACE2* and *TMPRSS2* at the resection margins of lung cancer patients as well as in normal tissues of non-cancer individuals to investigate the susceptibility of lung cancer patients to COVID-19. In addition, we retrospectively collected and analyzed detailed clinical data from lung cancer patients with laboratory-confirmed COVID-19 infection at the Wuhan Huoshenshan Hospital to help clinicians with the accurate treatment.

## Materials and Methods

### Microarray Data Analysis

The sequencing data of resection margin tissues from lung cancer patients were obtained from The Cancer Genome Atlas (TCGA) database and included 59 LUAD patients and 51 lung squamous cell carcinoma (LUSC) patients. Data on normal lung tissues from the general population were obtained from the GTEx database (https://gtexportal.org/home/) (n = 288). Phosphoglycerate kinase 1 (*PGK1*) was used for normalization between two different databases.

### Single-Cell RNA-Sequencing Data Analysis

The single-cell RNA-sequencing data of resection margin tissues and normal lung tissues were obtained from existing studies (11) and subjected to analysis *via* the Seurat R package (version 3.0, https://satijalab.org/seurat/). *EPCAM* and *IDH1* were used to identify epithelial cells. The copy number score of cells was predicted using the inferCNV R package (https://github.com/broadinstitute/infercnv).

### Immunohistochemistry (IHC)

After surgery, lung tissues obtained from patients with lung cancer or benign lung disease were prepared as formalin-fixed paraffin-embedded sections. Samples were cut into 4-μm-thick serial sections, deparaffinized with xylene, and rehydrated in alcohol. The samples were subsequently submerged in an antigen retrieval buffer and microwaved for antigen fixation. Sections were treated with hydrogen peroxide to block endogenous nonspecific binding activity and incubated for overnight at 4°C with diluted primary antibodies. Slides were incubated with appropriate horseradish peroxidase-conjugated secondary antibodies at 37°C for 1 h. Rabbit anti-cytokeratin 19 (CK19) antibody (Abcam, Cambridge, UK) was used as the primary antibody. Negative control slides were treated as per the same protocol except that the primary antibody was replaced with phosphate-buffered saline (PBS).

### Study Population and Data Collection

We carried out a retrospective case study at the Wuhan Huoshenshan Hospital, which was specially built to treat patients infected with COVID-19. Between 4 February and 11 April, 2020, 13 patients previously diagnosed with lung cancer and with laboratory-confirmed COVID-19 were enrolled. We used propensity score matching methods to select 52 patients as a control group with appropriate controls for other factors (e.g., age, sex, and comorbidities) to investigate the impact of lung cancer on COVID-19 as an independent factor. MatchIt function of R was used to achieve it, and the covariates are gender, age, and comorbidities, such as hypertension, diabetes, with ratio = 4. Clinical data were extracted from the hospital electronic medical records, including demographic features, clinical symptoms, laboratory and chest computed tomography (CT) results, treatments, and outcomes. This study was approved by the Medical Ethical Committee of Wuhan Huoshenshan Hospital. Written informed consent was obtained from each patient.

### Study Definitions

The severity of COVID-19 was evaluated as per the Seventh Revised Trial Version of the Novel Coronavirus Pneumonia Diagnosis and Treatment Guidance. (http://www.nhc.gov.cn/yzygj/s7652m/202003/a31191442e29474b98bfed5579d5af95.shtml) (8). Based on the sixth grade scale score proposed by Cao (http://www.chictr.org.cn/showproj.aspx?proj=49081) (12), time for clinical symptom remission was defined as the patient’s admission status as “discharged” or “a score reduction by two points.” Lung cancer patients with a survival time of more than 3 years were defined as medium-term survivors.

### Statistical Analysis

For descriptive analysis, continuous variables were summarized as medians and interquartile range (IQR), and categorical variables, as counts and percentages. The Wilcoxon rank-sum test or Fisher’s exact test was used to compare differences between groups, as appropriate. All statistical analyses were performed using SPSS Statistics Version 24.0 (IBM, New York, NY) and R studio (Version 1.2.1335; R Studio, Inc.). A two-sided value of *P <*0.05 was considered statistically significant.

## Results

### *ACE2* and *TMPRSS2* Are Overexpressed at Resection Margins of Lung Cancer Patients

**Figure 1:**
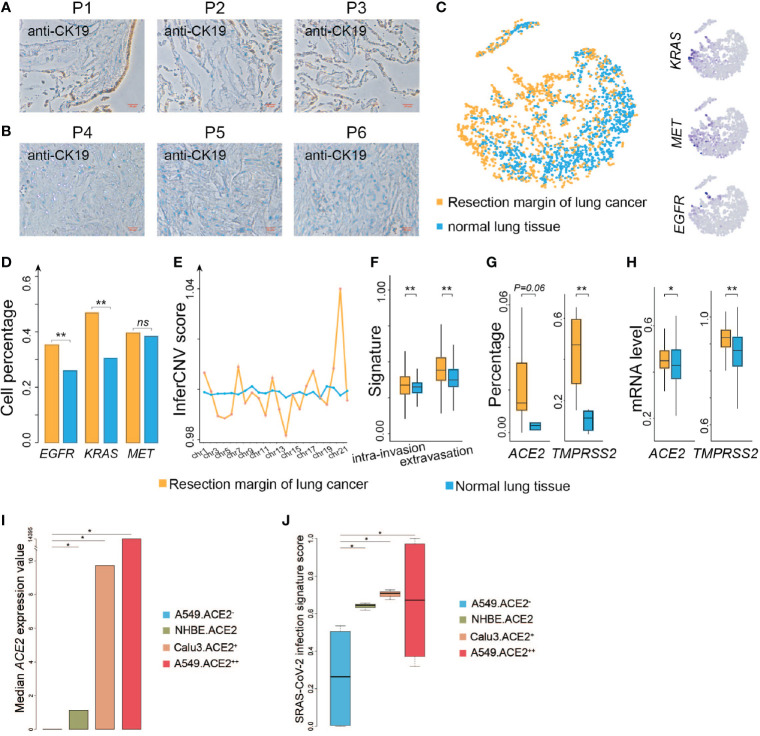
*ACE2* and *TMPRSS2* are highly expressed at resection margins of lung cancer patients. **(A)** Immunohistochemistry images of resection margin tissues from lung cancer patients using anti-CK19 antibody. **(B)** Immunohistochemistry images of non-cancer samples using anti-CK19 antibody. **(C)** Left: t-SNE plot of epithelial cells from the resection margins of lung cancers and normal lung tissues. Right: Distribution of the indicated cell marker genes overlaid on a 2D-tSNE plot. **(D)** Comparison of the percentage of indicated genes expressed in epithelial cells between the resection margin of lung cancers and normal lung tissues. **(E)** Comparison of inferCNV scores of cells between the resection margin of lung cancers and normal tissues across indicated chromosomes. **(F)** Comparison of intra-invasion and extravasation signature scores between indicated groups. **(G)** Comparison of the percentage of *ACE2* and *TMPRSS2* expressed in cells between indicated groups. **(H)** Comparison of mRNA levels of *ACE2* and *TMPRSS2* between indicated groups. **(I)** Bar plot shows median value of *ACE2* expression in different cell lines. A549.ACE2-, none *ACE2* expressed SARS-Cov-2 infected A549 cell lines; NHBE.ACE2, low *ACE2* expressed SARS-CoV-2 infected primary human lung epithelium cell lines; Calu3.ACE2+, median *ACE2* expressed SARS-Cov-2 infected Calu3 cell lines; A549.ACE2++, high *ACE2* expressed SARS-Cov-2 infected A549 cell lines. **(J)** Boxplot shows SARS-Cov-2 infection signature score in different cell lines as shown in **(I)**. *P < 0.05 (Wilcoxon rank sum test), **P < 0.01 (Wilcoxon rank sum test), NS, not significant.

Several studies have shown that coronaviruses enter cells *via* binding of the viral spike (S) proteins to cellular receptors *ACE2* and following S protein priming by host cell proteases. *TMPRSS2* as a transmembrane protease can induce the virus-plasma membrane fusion (13, 14). Hoffmann and coworkers recently demonstrated that SARS-CoV-2 uses the SARS-CoV receptor *ACE2* for entry and the serine protease *TMPRSS2* for S protein priming (15, 16). *ACE2* is broadly expressed in epithelial cells (6). We then evaluated if resection margins of lung cancer harbor more epithelial cells. We collected three resection margin tissues from lung cancer patients who underwent surgical treatment at Jiangsu Provincial Hospital in May 2020 and three other non-cancerous lung tissues. The surgical pathological stages of these three patients were all stage I, and so far, no tumor recurrence has occurred in the three patients (detailed information of control patients is listed in **Table S1**). We performed IHC to investigate the population of epithelial cells among the two groups, and found that the percentage of the stained area was much higher in the resection margin tissues than in the non-cancerous tissues (**Figures 1A, B**). We analyzed single-cell sequencing data from the existing research (11) and found that the epithelial cells at the resection margin of lung cancer were more likely to highly activate the genes related to lung cancer, such as *KRAS*, *MET*, and *EGFR* (**Figures 1C, D**). The genomic instability of these cells inferred by inferCNV (see *Materials and Methods*) in the resection margin was much higher than that in the normal tissues (**Figure 1E**), and these cells had stronger capability for invasion and infiltration (**Figure 1F**). These findings suggested that the cells at the margin of resection were more likely to be a sort of tumor-like cells.

The proportion of epithelial cells expressing *ACE2* and *TMPRSS2* was higher at the resection margin of lung cancers than in normal tissues (**Figure 1G**), suggesting that the resection margins of lung cancer tissues were still more susceptible to COVID-19 infection. We also analyzed the expression of *ACE2* in resection margin tissues of lung cancer survivors and normal lung tissues from general individuals using TCGA (n = 110) and GTEx (n = 288) databases and found that the mRNA expression of *ACE2* was higher in lung cancer patients than in general individuals (Wilcoxon’s rank-sum test, *P* = 0.013) (**Figure 1H**). The expression of *TMPRSS2*, the co-factor of *ACE2*, was also significantly higher in lung cancer patients than in the general population (Wilcoxon’s rank-sum test, *P <* 0.001) (**Figure 1H**), suggesting that patients with lung cancer were more likely to be susceptible to COVID-19.

To further verify our findings, we compare the median value of *ACE2* expression in different SARS-Cov-2 infected cell lines from GSE147507 (17) (**Figure 1I**). And we found the score of the signature SARS-Cov-2 infection identified by Li et al. (18) is significantly higher in *ACE2* high expressed cell lines than *ACE2* low expressed cell lines (Student’s t-test, *P <* 0.05) (**Figure 1J**), indicating high *ACE2* expressed cells may be more susceptible to SARS-Cov-2, suggesting that patients with lung cancer were more likely to be susceptible to COVID-19.

### Clinical Characteristics and Outcomes of Lung Cancer Patients With COVID-19 Infection

Records of 3,057 patients with confirmed COVID-19 infection were collected from the Wuhan Huoshenshan Hospital between February 4 and April 11, 2020. Thirteen patients (0.43%) who suffered from lung cancer and 52 matched patients were enrolled (see *Materials and Methods*). The demographic and clinical features of these patients are shown in **Table 1**. The median (IQR) age of lung cancer patients was 65 (63–72) years, and 10 (76.9%) of them were men. The most common comorbidities were hypertension and diabetes observed in 30.8% patients. No significant differences were found in age, sex, and main symptoms and signs between the case and control groups. Further, the most prevalent symptom among the 65 enrolled patients was fever (n = 50, 76.9%), followed by cough (n = 41, 63.1%), fatigue (n = 30, 46.2%), and shortness of breath (n = 26, 40.0%).

**Table 1:** Characteristics of patients with COVID-19.

Characteristic	All patients (*n* = 65)	Lung cancer (*n* = 13)	Non-lung cancer (*n* = 52)	*P* value
**Age, years**	66 (56–72)	65 (63–72)	66 (56–72)	0.761
**Sex**				0.779
Male	48 (73.8%)	10 (76.9%)	38 (73.1%)	
Female	17 (26.2%)	3 (23.1%)	14 (26.9%)	
**Comorbidities**				
Any	38 (58.5%)	8 (61.5%)	30 (57.7%)	0.803
Hypertension	22 (33.8%)	4 (30.8%)	18 (34.6%)	0.795
Diabetes	17 (26.2%)	4 (30.8%)	13 (25.0%)	0.674
Chronic obstructive pulmonary disease	3 (4.6%)	1 (7.7%)	2 (3.8%)	0.557
Coronary heart disease	3 (4.6%)	1 (7.7%)	2 (3.8%)	0.557
Cerebrovascular disease	5 (7.7%)	1 (7.7%)	4 (7.7%)	1.000
Chronic liver disease	4 (6.2%)	3 (23.1%)	1 (1.9%)	0.005
**Symptoms and signs**				
Fever	50 (76.9%)	9 (69.2%)	41 (78.8%)	0.465
Chill	13 (20.0%)	2 (15.4%)	11 (21.2%)	0.644
Chest pain	1 (1.5%)	0 (0.0%)	1 (1.9%)	0.617
Cough	41 (63.1%)	8 (61.5%)	33 (63.4%)	0.257
Fatigue	30 (46.2%)	4 (30.8%)	26 (50.0%)	0.217
Shortness of breath	26 (40.0%)	5 (38.5%)	21 (40.4%)	0.126
Chest tightness	7 (10.8%)	3 (23.1%)	4 (7.7%)	0.112
Expectoration	10 (15.4%)	3 (23.1%)	7 (13.5%)	0.394
Dyspnea	3 (4.6%)	2 (15.4%)	1 (1.9%)	**0.040**
Diarrhea	3 (4.6%)	1 (7.7%)	2 (3.8%)	0.557
Headache	3 (4.6%)	0 (0.0%)	3 (5.8%)	0.379
Myalgia	12 (18.5%)	3 (23.1%)	9 (17.3%)	0.634
Nausea	1 (1.5%)	0 (0.0%)	1 (1.9%)	0.617
Vomiting	2 (3.1%)	0 (0.0%)	2 (3.8%)	0.476

Bold value of p-value means P < 0.05.

One out of the thirteen patients died (7.7%) due to severe infection caused by COVID-19, while the remaining twelve patients were cured and discharged from the hospital. Five out of the thirteen lung cancer patients survived for more than three years, these five patients did not receive any anti-tumor treatment within three months before and after infection with COVID-19. The remaining eight patients were diagnosed with lung cancer less than three years, two of them did not receive any anti-tumor treatment within three months before and after infection, and six were infected with COVID-19 during anti-tumor treatment (detailed information of patients is listed in **Table S2**).

Patients with lung cancer were more likely to have dyspnea (15.4% *vs*. 1.9%; *P* = 0.040) than the other groups. Five of these patients were medium-term survivors, and none of them was diagnosed with stage IV cancer. All patients received at least one kind of antitumor treatment, including surgery (*n* = 7, 53.8%), chemotherapy (*n* = 6, 46.2%), epidermal growth factor receptor tyrosine kinase inhibitor (EGFR-TKI) therapy (*n* = 2, 15.4%), anti-angiogenesis therapy (*n* = 1, 7.7%), and radiotherapy (*n* = 2, 15.4%), while none of the medium-term survivors received treatment within past 3 months. The percentage of medium-term survivors who underwent surgery was four-fold higher than that of short-term survivors (100.0% *vs*. 25.0%, *P* = 0.011). No difference was observed in comorbidities and symptoms between medium- and short-term survivors (**Table S3**).

During the course of treatment, development of severe infection was more common among lung cancer patients (Wilcoxon’s rank-sum test, *P* = 0.064) (**Table 2**). The duration of symptoms before hospital admission in lung cancer patients was 10.5 (10.0–17.5) days, which was significantly shorter than that observed in other patients [30.0 (14.0–35.0); Wilcoxon’s rank-sum test, *P* = 0.016] (**Table 2**). Moreover, the average time to clinical improvement in lung cancer patients was 12 (11.0–18.0) days, which was 4 days longer than that observed in non-cancer patients (5.8–14.0) (Wilcoxon’s rank-sum test, *P* = 0.020) (**Table 2**). There were no differences among indicators such as time from symptoms to hospitalization and symptom remission time between medium-term and short-term survivors (**Table 3**).

**Table 2:** Outcome of lung cancer patients and general population.

Characteristic	All (*n* = 65)	Lung cancer (*n* = 13)	Non-lung cancer (*n* = 52)	*P* value
**Hospital stay (days)**	11.0 (8.0–18.0)	13.0 (11.0–18.0)	10.0 (7.0–17.3)	0.178
**Most critical type during hospitalization**				0.064
Mild/Moderate	29 (44.6%)	3 (23.1%)	27 (51.9%)	
Severe/Critical	35 (53.8%)	10 (76.9%)	25 (48.1%)	
**Time from symptoms to hospitalization (days)**	19.0 (10.0–35.0)	10.5 (10.0–17.5)	30.0 (14.0–35.0)	**0.016**
**Clinical symptoms remission time (days)**	9.0 (6.0–15.0)	12.0 (11.0–18.0)	8.0 (5.8–14.0)	**0.020**
**Admission to intensive care unit**				0.433
Yes	4 (6.2%)	2 (15.4%)	2 (3.8%)	
No	61 (93.8%)	11 (84.6%)	50 (96.2%)	
**ICU stay (days)**	16.5 (13.5–21.5)	13.5 (11.3–15.8)	23.5 (19.3–27.8)	
**Clinical outcomes**				**0.046**
Discharge from hospital	64 (98.5%)	12 (92.3%)	52 (100.0%)	
Death	1 (1.5%)	1 (7.7%)	0 (0.0%)	
**Time from diagnosis to death (days)**	18.0 (18.0–18.0)	18.0 (18.0–18.0)	–	–

Bold value of p-value means P < 0.05.

**Table 3:** Outcome of medium-term and short-term lung cancer survivor.

Characteristic	Lung cancer (*n* = 13)	Medium-term survivor (*n* = 5)	Short-term survivor (*n* = 8)	*P* value
**Hospital stay (days)**	13.0 (11.0–18.0)	13.0 (12.0–19.0)	13.5 (10.5–16.5)	1.000
**Most critical type during hospitalization**				0.841
Mild/Moderate	3 (23.1%)	1 (20.0%)	2 (25.0%)	
Severe/Critical	10 (76.9%)	4 (80.0%)	6 (75.0%)	
**Time from symptoms to hospitalization (days)**	10.0 (10.0–16.0)	14.0 (10.0–16.0)	10.0 (9.3–15.8)	0.552
**Clinical symptoms remission time (days)**	12.0 (11.0–18.0)	12.0 (11.0–19.0)	13.0 (10.5–17.3)	1.000
**Admission to intensive care unit**				0.221
Yes	2 (15.4%)	0 (0%)	2 (25.0%)	
No	11 (84.6%)	5 (100%)	6 (75.0%)	
**ICU stay (days)**	13.5 (11.3–15.8)	–	13.5 (11.3–15.8)	–
**Clinical outcomes**				0.429
Discharge from hospital	12 (92.3%)	5 (100%)	7 (87.5%)	
Death	1 (7.7%)	0 (0%)	1 (12.5%)	
**Time from diagnosis to death (days)**	18.0 (18.0–18.0)	–	18.0 (18.0–18.0)	–

## Discussion

In this study, we show that lung cancer patients infected with SARS-COV-2 tend to have more severe outcomes as compared to the general population. The mortality was higher in the lung cancer cohort than in the control cohort and the difference was statistically significant, consistent with previous findings (19). However, the mortality (7.7%, 1/13) observed in our study was higher than that reported in the general population (2.3%) and lower than that (18.18%; 4/22) noted in a multicenter study (19, 20). Lung cancer patients seemed to be more likely to develop severe infection but without any significant difference (*P* = 0.064). This trend is consistent with that observed in previous studies (21–23). The median time from symptom onset to hospitalization was shorter and the clinical symptom remission time was longer in lung cancer patients infected with SARS-COV-2 than in the general population, indicating that the disease develops more rapidly in lung cancer patients. Thus, lung cancer patients represent a highly vulnerable group to the current COVID-19 outbreak.

Medium-term lung cancer survivors and short-term lung cancer patients had unexpectedly similar outcomes. Recent studies associated the increased risk of developing severe events in cancer patients to their systemic immunosuppressive state caused by antitumor treatments such as chemotherapy, radiotherapy, targeted therapy, and immunotherapy (21, 23). Our results seem to contradict those of previous studies, probably owing to the higher expression detected at the resection margin of lung cancer patients. The non-significance may not rule out the differences between medium-term and short-term lung cancer patients owing to the small sample size.

We also explored the differences in the expression of *ACE2* and *TMPRSS2* in resection margin tissues of lung cancer patients and normal lung tissues of non-cancerous patients. *ACE2* gene expression at the resection margin was higher than that in normal lung tissues, while *TMPRSS2* showed even higher expression. Given that elevated levels of *ACE2* and *TMPRSS2* may indicate higher susceptibility to SARS-CoV-2 (15), our findings show that lung cancer patients are more vulnerable to SARS-CoV-2. A recent study analyzed the expression of *ACE2* across over 30 tumors and reported *ACE2* overexpression in LUAD (7). Another study investigated the expression of *ACE2* and *TMPRSS2* genes in LUAD and LUSC and suggested higher and nearly equal *ACE2* expression in LUAD and LUSC tumor tissues than in normal tissues, respectively (8). These authors also profiled the expression of *ACE2* and *TMPRSS2* genes in each pathological stage of two lung cancer types and found consistent expression patterns in each pathological stage of lung cancer, suggesting equal susceptibility to SARS-CoV-2 among patients with different pathological stages of LUAD and LUSC (8). These two studies with consistent results imply that patients with lung cancer are more vulnerable to SARS-CoV-2 attacks. However, the expression of *ACE2* and *TMPRSS2* in resection margin tissues may be more valuable in medium-term lung cancer survivors.

The increase in the population of epithelial cells in resection margin tissues may be the possible mechanism that deems lung cancer patients highly susceptible to SARS-CoV-2. Previous studies have confirmed the high expression of *ACE2* and *TMPRSS2* in type II alveolar cells (AT2), an epithelial cell type (6, 24). Interestingly, our results show that epithelial cells were enriched in resection margin tissues and showed upregulated expression of *ACE2* and *TMPRSS2*. Despite cancer resection, the remaining tissue still retains the characteristics of cancer cells, such as genome instability and strong local infiltration and extravasation abilities. As *ACE2* and *TMPRSS2* are overexpressed in lung cancer (7, 8), their expression may be high at the resection margin of lung cancer patients. To our knowledge, this is the first study to compare the expression of *ACE2* and *TMPRSS2* in resection margin tissues of lung cancer patients and normal lung tissues from non-cancerous individuals. Our results may explain why medium-term lung cancer survivors are more susceptible to SARS-CoV-2 and highly vulnerable to the COVID-19 pandemic. These results indicate stronger personal protection not only for short-term lung cancer patients but also for medium-term survivors.

In our study, we found one out of thirteen (7.7%) median-term survival cancer patients died with the infection of SARS-COV-2, diagnosed with a series of infection symptoms, including fever, cough, shortness of breath, expectoration, dyspnea. Many studies show lung cancer patients have a higher mortality rate than the general population which is similar to our finding. For instance, Luo et al. (25) showed the COVID-19 infection associated with a high burden of severity in patients with lung cancer. 25 out of 102 (25%) patients died due to the progression of disease caused by the COVID-19 infection. Rogado et al. (26) retrospectively reviewed 1,878 medical records of all COVID-19 patients, finding that nine out of seventeen (52.3%) lung cancer patients died with COVID-19. Asymptomatic patients may influence the case fatality rate of lung cancer patients with COVID-19, nevertheless, many studies have showed lung cancer patients with COVID-19 were severer than general patients with COVID-19. Hence, we argue that our study indicates lung cancer patients with COVID-19 need to be carefully considered and shows the possible reason for lung cancer survivors are susceptible to SRAS-COV-2.

Although our study highlights the vulnerability of patients with lung cancer to the COVID-19 pandemic, it has some limitations. Since elevated levels of *ACE2* and *TMPRSS2* indicate a high susceptibility to SARS-CoV-2, and medium-term survivors did not have tumor burden, the expression of *ACE2* and *TMPRSS2* at resection margin tissue may indicate the susceptibility of lung cancer patients to SARS-CoV-2. So we detected the expression of *ACE2* and *TMPRSS2* at the resection margin of lung cancer patients to explore the molecular mechanism of lung cancer patients’ high susceptibility to SARS-CoV-2. However, we failed to obtain tissue specimens from lung cancer patients with laboratory-confirmed COVID-19 infection, hence, the results could not directly reflect the relationship between the level of SARS-CoV-2 receptor expression and the outcome of lung cancer survivors. Further, not all patients had complete information on immune-related indicators; therefore, the correlation between tumor immunity and SARS-CoV-2 in lung cancer survivors should be further explored. Previous studies have shown that *ACE2* attenuated the metastasis of lung cancer and that *TMPRSS2* fusion gene may induce resistance to EGFR-TKI, a standard first-line therapy for advanced NSCLC patients harboring EGFR mutation (27, 28). Little is known about the interaction between SARS-CoV-2 and lung cancer cells and how COVID-19 affects lung cancer patients. The molecular mechanism requires further exploration. Finally, the small sample size, retrospective nature were also limitations in our study.

## Conclusions

This study revealed the high expression of the SARS-CoV-2 receptors, *ACE2* and *TMPRSS2*, at resection margins of lung cancer survivors and its possible relationship with the higher susceptibility of these patients to COVID-19. Clinical data revealed that lung cancer patients, including medium-term survivors, diagnosed with COVID-19 infection may have worse outcomes and should be carefully considered.

## Data Availability Statement

The original contributions presented in the study are publicly available. This data can be found here: https://www.ebi.ac.uk/arrayexpress/experiments/E-MTAB-6149/, https://www.ebi.ac.uk/arrayexpress/experiments/E-MTAB-6653/, and https://www.tissuestabilitycellatlas.org/.

## Ethics Statement

This study was approved by the Medical Ethical Committee of Wuhan Huoshenshan Hospital. Written informed consent was obtained from each patient. The patients/participants provided their written informed consent to participate in this study.

## Author Contributions

XX, PL, ZhiZ, and RG have full access to all of the data in the study and take responsibility for the integrity of the data and the accuracy of the data analysis. QQW, LL, TQ, and JL contributed equally. Concept and design: XX, PL, ZhiZ, and RG. Data collection: XX, QHW, TQ, and ZZ. Data analysis and interpretation: LL, JL, LW, KL, ZW, MZ, BH, WW, MW, and RD. Drafting of the manuscript: QQW, LL, TQ, and JL. All authors contributed to the article and approved the submitted version.

## Funding

This work was supported by National Natural Science Foundation of China (NSFC 81972188, 81572893, 81972358, 81959113) and the Medical Important Talents (ZDRCA2016024).

## Conflict of Interest

The authors declare that the research was conducted in the absence of any commercial or financial relationships that could be construed as a potential conflict of interest.

## Supplementary Material

Supplementary Table 1 Characteristics of lung cancer patients with COVID-19.

Supplementary Table 2 Characteristics of patients with donated tissues.

Supplementary Table 3 The specific treatment and stage for each patient.

## Abbreviations

COVID-19, Coronavirus disease 2019; SARS-COV-2, Severe acute respiratory syndrome coronavirus 2; MERS, Middle East respiratory syndrome; ACE2, Angiotensin-converting enzyme 2; TMPRSS2, Transmembrane protease serine 2; LUAD, Lung adenocarcinoma; NSCLC, Non-small cell lung cancer; CT, Computed tomography; TCGA, The Cancer Genome Atlas; PGK1, Phosphoglycerate kinase 1; IQR, Interquartile range; AT2, Type II alveolar cells.
